# Activation of the calcium-sensing receptor by glutathione maillard products: Implications for kokumi sensation

**DOI:** 10.1016/j.fochx.2025.102616

**Published:** 2025-06-03

**Authors:** Raise Ahmad, Santanu Deb-Choudhury, Arvind Subbaraj, Jihan Kim

**Affiliations:** a AgResearch, Smart Foods & Bioproducts Group, Palmerston North, New Zealand; b AgResearch, Smart Foods & Bioproducts Group, Lincoln, New Zealand

**Keywords:** Aroma, Browning, Flavour, Kokumi, Receptor, Taste, Volatile

## Abstract

Kokumi peptides, such as glutathione (GSH), activate the calcium-sensing receptor (CaSR) in taste buds, evoking distinct sensory attributes. Foods containing GSH-derived Maillard reaction products (MRPs) often exhibit kokumi characteristics, but their direct interaction with CaSR remains unclear. In this study, GSH was thermally reacted with reducing sugars (glucose, xylose) at 80 °C, 120 °C, and 150 °C. The resulting MRPs were evaluated for browning, volatile profiles (GC–MS), and CaSR activation using a fluorescence-based cell assay. GSH–xylose MRPs showed greater browning and distinct volatile profiles compared to GSH–glucose MRPs. Notably, GSH–glucose MRPs showed highest CaSR activity at >120 °C, followed by GSH–xylose MRPs, likely acting as positive allosteric modulators. This provides the first evidence of allosteric modulation of CaSR by GSH–sugar MRPs, offering mechanistic insight into receptor interaction. However, the lack of sensory validation remains a limitation, and further studies are needed to confirm the perceptual relevance of these findings.

## Highlights

• GSH-sugar Maillard products enhance CaSR activation response.
• Thermal processing and Maillard reaction impact CaSR activation.
• GSH degradation reduces CaSR activation, glucose enhances it.
• Xylose affects aromatic compounds more than CaSR activation.
• GSH and glucose in cooked foods offer flavour enhancement potential.

## Graphical abstract

**Figure:**
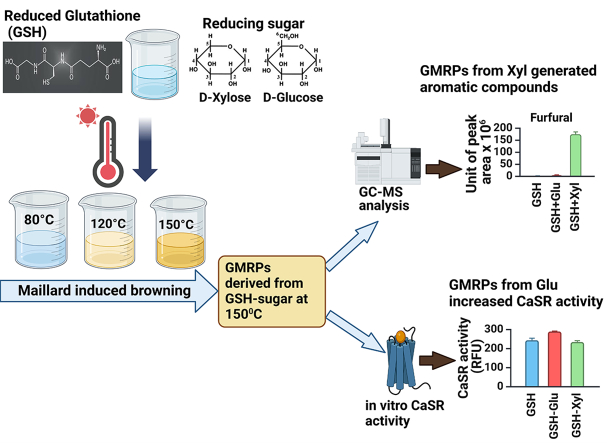

## 1 Introduction

The Maillard reaction between amino acids and reducing sugars generates a complex mixture of compounds responsible for savoury and roasted flavours, enhancing the depth and richness of foods (Starowicz & Zieliński, 2019; Van Boekel, 2006). Some of these Maillard reaction products (MRPs) are associated with kokumi, a flavour attribute that imparts continuity, richness and complexity to taste (Xu, You, Song, Gong, & Pan, 2018). Although MRPs from protein hydrolysates have been linked to kokumi characteristics (L. Kang, Alim, & Song, 2019; Zhu et al., 2023), the biochemical mechanisms underlying their kokumi-enhancing properties remain poorly understood.

Glutathione (GSH), a γ-glutamyl-cysteinyl-glycine tripeptide naturally present in garlic, onions, and meat, is known to contribute to kokumi by generating sulphur-containing compounds during thermal processing (Kwon, Hong, Kim, Lee, & Kim, 2011). However, GSH is thermally unstable and prone to degradation at high temperatures, which may impact its sensory functionality. While native GSH is a known kokumi enhancer, the contribution of its thermally resulted volatile and non-volatile MRPs to kokumi taste perception has received little attention.

Studies have demonstrated that kokumi peptides, including GSH, activate the calcium-sensing receptor (CaSR) on taste bud cells, contributing to kokumi sensory attributes such as continuity, mouthfullness, thickness, and enhanced flavour (Ahmad & Dalziel, 2020; Maruyama, Yasuda, Kuroda, & Eto, 2012; Ohsu et al., 2010). As a result, CaSR activation has become a reliable in vitro indicator for identifying potential kokumi-active compounds. However, the ability of MRPs particularly those formed from GSH and different reducing sugars to activate CaSR or function as allosteric modulators remains poorly understood. To date, no studies have investigated whether MRPs generated from GSH in combination with specific sugars such as glucose or xylose can modulate CaSR activity.

To address this, we investigated the effects of thermal treatment and Maillard reactions between GSH and two reducing sugars (glucose and xylose) at varying temperatures (80 °C, 120 °C, and 150 °C) on CaSR activation in vitro. We also analysed the browning intensity and volatile compound profiles of the resulting MRPs to better understand their chemical transformations. We hypothesise that MRPs, particularly those generated at elevated temperatures can act as allosteric modulators of CaSR, thereby enhancing its activity. This study provides novel insights into the molecular basis of kokumi enhancement by MRPs, laying the groundwork for their application in thermally processed food products.

## 2 Methods

### 2.1 Experimental design

**Fig. 1:**
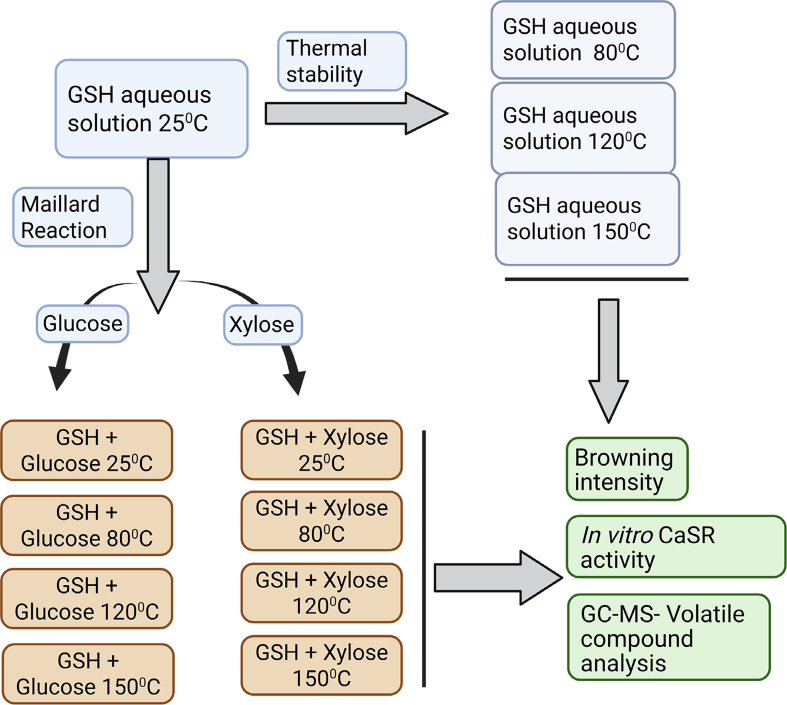
Schematic representation of experimental methodology used in the study (created with Biorender.com).

GSH, xylose and glucose were purchased from Sigma-Aldrich. The experimental design (Fig. 1) illustrates GSH thermal stability and Maillard reaction with sugars, including analysis of volatiles and CaSR activity by measuring intracellular calcium levels. GSH solution (0.01 M) in water was heated at 80, 120, and 150 °C, compared against ambient temperature (25 °C). These three temperatures were chosen to represent specific cooking processes: slow cooking at 80 °C, roasting at 120 °C and high-temperature roasting at 150 °C (Kim, Deb-Choudhury, Subbaraj, Realini, & Ahmad, 2024). All samples were prepared as three biological replicates, each with technical triplicates.

### 2.2 Maillard reaction of GSH and sample preparation

GSH (0.01 M) and d-glucose or D-xylose (0.01 M) were dissolved in 10 mL of Milli-Q water, and the pH was adjusted to 7 using 1 M HCl and 1 M NaOH. The solutions were then sealed in glass tubes with screw cap (18 × 180 mm). Samples were heated at 80, 120, and 150 °C in silicon oil bath for 3 h, then cooled to 4 °C overnight to allow the pressure inside the tubes to normalise and to stabilise the reaction products.

### 2.3 Cell culture and maintenance

Mammalian cell lines, CHO-K1-CaSR (stably expressing human CaSR; #M00434, GenScript, NJ, USA) and CHO-K1-Gα15 (stably expressing Gα15 protein; #M00257, GenScript) were cultured in Ham's F12 Glutamax media (#31765035, Gibco, TX, USA), supplemented with 10 % foetal bovine serum, 1 % penicillin-streptomycin, and selection antibiotics zeocin (200 μg/mL) for CHO-K1-CaSR and hygromycin (100 μg/mL) for CHO-K1-Gα15. The cells were maintained at 37 °C in a 5 % CO₂ incubator. The passage number of the cells used for all assays ranged from 3 to 5, and cells were seeded at a confluency of approximately 80 % prior to experimentation. On the day before the experiment, 70,000 cells/well were seeded in 96-well plates, following optimized conditions for maximal CaSR responsiveness based on previous studies (Ahmad et al., 2023; Kim et al., 2022; Kim, Deb-Choudhury, Subbaraj, Realini, & Ahmad, 2024). These conditions were chosen to ensure optimal receptor expression and functionality, providing reliable CaSR activation in response to stimuli.

### 2.4 In vitro CaSR activity assay

Calcium-sensing receptor (CaSR) activation was assessed using the FLIPR® Calcium 6 Assay Kit (#R8191, Molecular Devices, San Jose, CA, USA), which contains a proprietary calcium-sensitive dye functionally similar to Fluo-4 AM. The assay was conducted according to the manufacturer's instructions and based on protocols optimized in previous studies (Ahmad et al., 2023; Kim et al., 2022; Kim et al.; 2024).

CHO-K1 cells stably expressing human CaSR and CHO-K1-Gα15 control cells (lacking CaSR expression) were seeded in black-walled, clear-bottom 96-well plates and incubated overnight at 37 °C with 5 % CO₂. Cells were then loaded with FLIPR Calcium 6 dye in loading buffer and incubated for 2 h at 37 °C, as validated in our previous optimization studies to ensure consistent dye uptake and a stable baseline fluorescence. This duration was optimized to achieve the highest signal-to-noise ratio, reduce well-to-well variation, and support assay reproducibility as published in our previous studies (Ahmad et al., 2023; Kim et al., 2022; Kim et al.; 2024).

Total peptide concentrations of GSH alone or Maillard reaction samples were quantified using quantitative fluorometric peptide assay kit (#23290; Pierce-Thermo Scientific) and normalized to 2.0 mg/mL, and serially diluted two-fold in assay buffer to prepare seven concentrations: 0.8, 0.4, 0.2, 0.1, 0.05, 0.025, and 0.00625 mg/mL. 100 μL of each dilution was added to cells in triplicate (*n* = 3 biological replicates per treatment). The 96-well assay plate was organized to include CaSR-expressing CHO-K1 cells treated with each dilution of the experimental samples, alongside CHO-K1-Gα15 cells (which do not express CaSR) to assess non-specific responses. Additionally, buffer-only wells containing dye only were included to determine background fluorescence. Intracellular calcium flux was measured using a FlexStation® 3 microplate reader (excitation: 485 nm; emission: 525 nm). The resulting relative fluorescence units (RFU) were baseline-corrected by subtracting buffer-only background from each sample signal. These values (ΔRFU) reflect receptor-specific calcium responses.

### 2.5 Browning intensity

Browning intensity was measured using a UV–Vis spectrophotometer (M. Yu et al., 2018), with absorbance at 420 nm as an index of brown polymers.

### 2.6 Analysis of volatile compounds by GC–MS

Volatile compounds in the sample headspace were extracted using SPME and analysed via GC–MS. Samples were heated at 40 °C for 30 min with agitation. The SPME fiber was exposed to the headspace for 30 min, then desorbed in a split-less injector. Chromatographic separation was performed using a Shimadzu QP-2010 Plus GC system, and mass spectra were acquired using a Shimadzu TQ 8040 mass spectrometer. Peaks were analysed using the NIST14 database (≥90 % similarity). For further method details and identification of sulphur compounds please see supplementary material.

### 2.7 Analysis of GSH by LC-MS

GSH control and heat-treated samples were analysed using HILIC LC-MS in negative ionization mode (Kim et al., 2022). Peak areas were compared as relative values. For further details please see supplementary material.

### 2.8 Statistical analysis

Data were analysed using GraphPad Prism 10. ANOVA with Tukey's post-hoc test determined statistical significance (*p* < 0.05). Two-way ANOVA assessed temperature and sugar effects on browning intensity, while one-way ANOVA evaluated the impact of sugars on volatile peak intensities. Pearson correlation coefficients were calculated to evaluate the relationships between browning intensity, total volatile peak area, and CaSR activation (measured as relative fluorescence units, RFU) at 0.8 mg/mL. Correlations were considered significant at *p* < 0.05.

## 3 Results and discussion

### 3.1 Browning intensity of GSH during thermal processing

The development of brown colour is a visual indicator of the Maillard reaction, with absorbance at 420 nm commonly used to assess browning during advanced stages (Laroque et al., 2008). In this study, we analysed the browning of GSH with and without glucose and xylose at temperatures of 80 °C, 120 °C, and 150 °C for three hours. These temperatures represent typical cooking ranges relevant to the thermal reaction between sugars and protein hydrolysates (Eric et al., 2014). Results were compared to a control GSH solution (Con) at 25 °C.

**Fig. 2:**
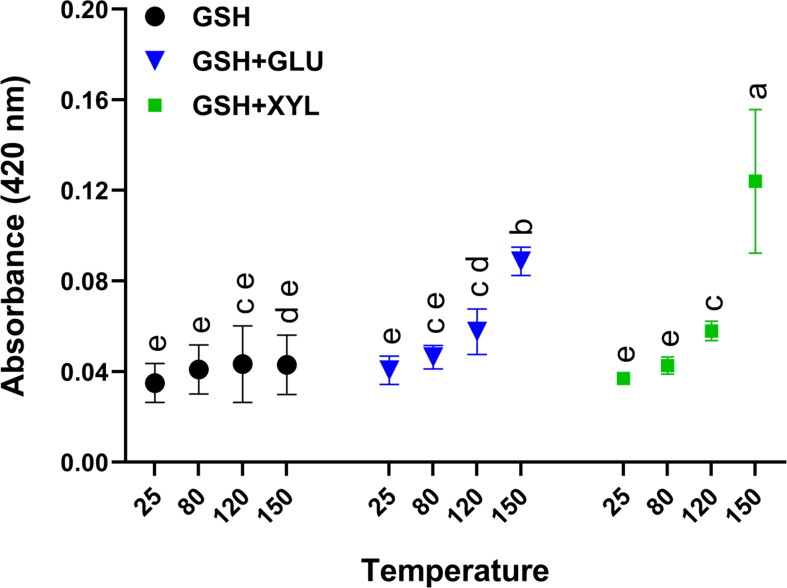
Browning intensity presented as absorbance of glutathione (GSH) alone or with reducing sugars glucose (GSH + GLU) or xylose (GSH + XYL) in aqueous solution, measured after 3 h incubation at ambient temperature (25 °C) and at elevated temperatures (80 °C, 120 °C, 150 °C). Data are shown as mean ± SEM, based on three independent experiments (*n* = 3). Treatments annotated with different letters are significantly different (*p* < 0.05). Statistical test: One-way ANOVA with Tukey's post hoc test.

When heated alone, GSH showed negligible change in absorbance at 420 nm. However, in the presence of glucose and xylose at 150 °C, browning intensity increased significantly (*p* < 0.01, Fig. 2). GSH-xylose exhibited a significantly greater increase in browning (p < 0.01) compared to GSH-glucose at 150 °C, indicating that elevated temperature was critical for promoting extensive Maillard reaction product formation. Given xylose higher reactivity compared to glucose (Alonso-Riaño et al., 2024), it was more effective in driving the Maillard reaction.

At 150 °C, both sugars reacted with GSH, but GSH-xylose showed significantly higher browning intensity than GSH-glucose (*p* < 0.05), suggesting more extensive Maillard reaction progression with xylose. We then investigated whether the difference in browning intensity led to variations in aromatic volatile profile and CaSR activity.

### 3.2 Composition of volatile analytes generated by GSH Maillard reaction with reducing sugars

The Maillard reaction of GSH at high temperatures primarily generates volatile flavour compounds that significantly affect the aroma and overall flavour of foods (Lee, Jo, & Kim, 2010). We investigated how GSH, both alone and in reaction with either glucose or xylose, generated these volatile compounds during thermal processing. A total of 55 volatile compounds were identified (Supplementary Table 1), with the Maillard reaction-derived volatile compounds extracted and presented in Table 1. Dimethyl disulfide (DMS) and dimethyl trisulfide (DMTS) are separately presented in Table 2 due to their significant contribution to the sulphurous aroma profile, which is a key characteristic in many thermally processed foods. These compounds, formed through the breakdown of GSH during the Maillard reaction, play a crucial role in shaping the overall flavour and aroma (Jensen, Hansen, & Andersen, 2002).

**Table 1:** Maillard reaction-derived volatile compounds (expressed in arbitrary units (au) of peak area × 10^6^) generated following GSH thermal processing alone as control (GSH) or with glucose or xylose at ambient (25 °C) and three different temperatures (80 °C, 120 °C, and 150 °C). Data are presented as Mean ± SD, *n* = 3. Means within a row not sharing letters are significantly different (p < 0.05, Tukey's test). ND, not detected.

	Temp (°C)	GSH	GSH + Glucose	GSH + Xylose
2,3-Butanedione	25	2.9 ± 0.8^b^	5.3 ± 0.2^a^	4.6 ± 0.8^a^
	80	ND	ND ND	2.3 ± 0.2
	120	ND		ND ND
	150	ND	2.5 ± 0.2	

2,3-Pentanedione	25	ND	ND ND ND ND	ND ND ND
	80	ND		
	120	ND		
	150	ND		12.1 ± 1.4

2-Furancarboxaldehyde, 5-methyl	25	ND ND ND ND	ND ND ND 1.9 ± 0.3	ND ND ND ND
	80			
	120			
	150			

2-Furanmethanol	25	ND	ND ND ND ND	ND ND ND
	80	ND ND ND		
	120			
	150			0.4 ± 0.0

2-Thiophenecarboxaldehyde	25	ND ND ND ND	ND	ND ND ND
	80		ND	
	120		ND	
	150		0.5 ± 0.0^b^	6.8 ± 0.9^a^

Furfural	25	0.2 ± 0.0	0.5 ± 0.2	0.2 ± 0
	80	0.3 ± 0.1^b^	0.9 ± 0.6^ab^	2.4 ± 1.8^a^
	120	0.5 ± 0.2^b^	0.5 ± 0.1^b^	19 ± 1.5^a^
	150	0.5 ± 0.1^c^	3.7 ± 0.4^b^	173. 8 ± 1.6^a^

Ethanone, 1-(2-furanyl)-	25	ND ND ND ND	ND ND ND	ND ND ND
	80			
	120			
	150		1.0 ± 0.2^a^	0.4 ± 0.2^b^

Thiazole	25	ND ND ND ND	ND ND ND	ND ND ND
	80			
	120			
	150		1.6 ± 0.2	1.1 ± 0.3

Thiophene, 3-methyl-	25	ND ND ND	ND ND ND	ND ND ND
	80			
	120			
	150	0.4c ± 0.0	8.5 ± 1.0^b^	43.4 ± 15.4^a^

Toluene	25	ND ND ND ND	ND ND ND	ND ND ND
	80			
	120			
	150		0.8 ± 0.1^b^	1.8 ± 0.8^a^

**Table 2:** Maillard reaction-derived Dimethyl disulfide and Dimethyl trisulfide volatile compounds (expressed in au of peak area × 10^6^) generated following GSH thermal processing or with glucose or xylose at ambient, 25 °C and three different temperatures (80 °C, 120 °C, and 150 °C). Data are presented as Mean ± SD, n = 3. Values within a row not sharing letters are significantly different (p < 0.05, Tukey's test). ND, not detected.

	Temp (°C)	GSH	GSH + Glucose	GSH + Xylose
Dimethyl trisulfide	25	5.0 ± 3.7^a^	1.8 ± 0.5^ab^	0.6 ± 0.3^b^
	80	0.7 ± 0.4	ND	ND
	120	4.6 ± 1.2^b^	11.1 ± 6.1^a^	17.6 ± 12.3^a^
	150	7.4 ± 2.0	ND	ND

Dimethyl disulfide	25	9.1 ± 6.3^a^	5.6 ± 1.7^b^	2.1 ± 1.2^b^
	80	2.5 ± 1.4^a^	0.4 ± 0.1^b^	ND
	120	10.9 ± 1.6	27.2 ± 15.1	27.4 ± 21.7
	150	10.3 ± 2.2^a^	2.5 ± 0.0^b^	3.8 ± 1.7^b^

At 150 °C, GSH combined with xylose led to a significant increase (*p* < 0.001) in the production of furfural and 3-methyl thiophene compared to the reaction with glucose (Table 1). Furfural is known for its almond-like aroma, and 3-methyl thiophene contributes to strong sulphurous aromas (X. Kang, Wang, Wang, & Song, 2021). They both began forming at high temperature of 150 °C only (Table 1) indicating that xylose is more reactive than glucose at high temperatures as suggested previously (Laroque et al., 2008). The presence of 3-methylthiophene suggests that sulphur-containing intermediates, potentially including free cysteine released from GSH degradation may have contributed to its formation, particularly in the xylose reaction. Overall, these results suggest that the GSH–xylose Maillard reaction promotes greater volatile compound formation than the glucose system, which may favour pathways yielding non-volatile products.

Table 2 highlights the formation of DMS and DMTS, which are sulphurous compounds with potent odours, often described as resembling cooked cabbage or onions. These compounds formed from GSH decomposition, where cysteine breaks down into hydrogen sulphide, then reacts with methyl groups from sugars to form methanethiol (T. Yu & Ho, 1995). Methanethiol further oxidizes to produce DMS and DMTS (Zhang, Wang, & Cao, 2023). These sulphurous compounds were prominent at 120 °C when reducing sugars were added. However, as the temperature increased from 120 °C to 150 °C, the sulphurous compounds in both Glucose-GSH and Xylose-GSH mixtures decreased, whereas those in GSH alone remained unchanged. This indicates that reducing sugar addition significantly influenced the formation and degradation of DMS and DMTS.

It was also observed that the levels of DMS and DMTS decreased at 80 °C compared to their levels at 25 °C, but increased when the temperature was raised to 120 °C to 150 °C. It has been reported that GSH degrades at 80 °C, with GSH-disulfide (GSSH) being the primary degradation product (Ueda, Yonemitsu, Tsubuku, Sakaguchi, & Miyajima, 1997). Therefore, the formation of hydrogen sulphide, a precursor for these sulphur compounds, is reduced due to the formation of GSSH, which likely leads to lower levels of DMS and DMTS. In summary, while GSH alone produced sulphurous compounds through cysteine breakdown, the addition of sugars notably altered the volatile compound profile. Although no direct evidence currently links DMS or DMTS to CaSR activation, their synergy with kokumi peptides observed in fermented foods hints at an indirect role in enhancing flavour complexity (Rodríguez Valerón, Mak, Jahn, Arboleya, & Sörensen, 2023). Future research should investigate their potential contribution to CaSR modulation. Their presence underscores key aroma–taste interactions that may enrich the kokumi sensory profile in our samples.

### 3.3 CaSR activity of GSH maillard reaction products with xylose and glucose

We used an in vitro cell-based assay to evaluate CaSR activation in response to GSH during thermal processing and its Maillard reaction with glucose and xylose. Dose-dependent responses were measured using CHO-K1 cells stably expressing CaSR. CHO-K1-Gα15 cells lacking CaSR were used as a negative control to confirm the specificity of receptor activation.

Serial dilutions of GSH samples were prepared based on total peptide concentration, ranging from 0.8 mg/mL to 0.0062 mg/mL, and applied to CaSR-expressing cells to measure intracellular calcium levels. The samples elicited a clear, concentration-dependent activation in CaSR-expressing cells, while control cells showed only minimal, non-specific fluorescence—even at the highest concentration of 0.8 mg/mL (Supplementary Fig. S1A—C). This confirms that the observed responses were specific to CaSR activation.

**Fig. 3:**
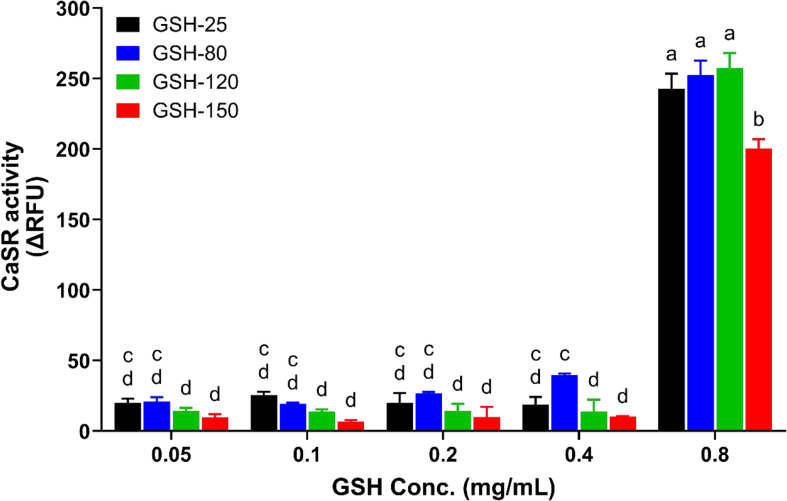
CaSR activation measured as relative fluorescence units (ΔRFU) in response to GSH thermally treated at 25 °C (GSH-25), 80 °C (GSH-80), 120 °C (GSH-120), and 150 °C (GSH-150). Data represent mean ± SEM from three independent experiments (n = 3). Treatments annotated with different letters are significantly different (p < 0.05). Statistical test: One-way ANOVA with Tukey's post hoc test.

**Fig. 4A, B:**
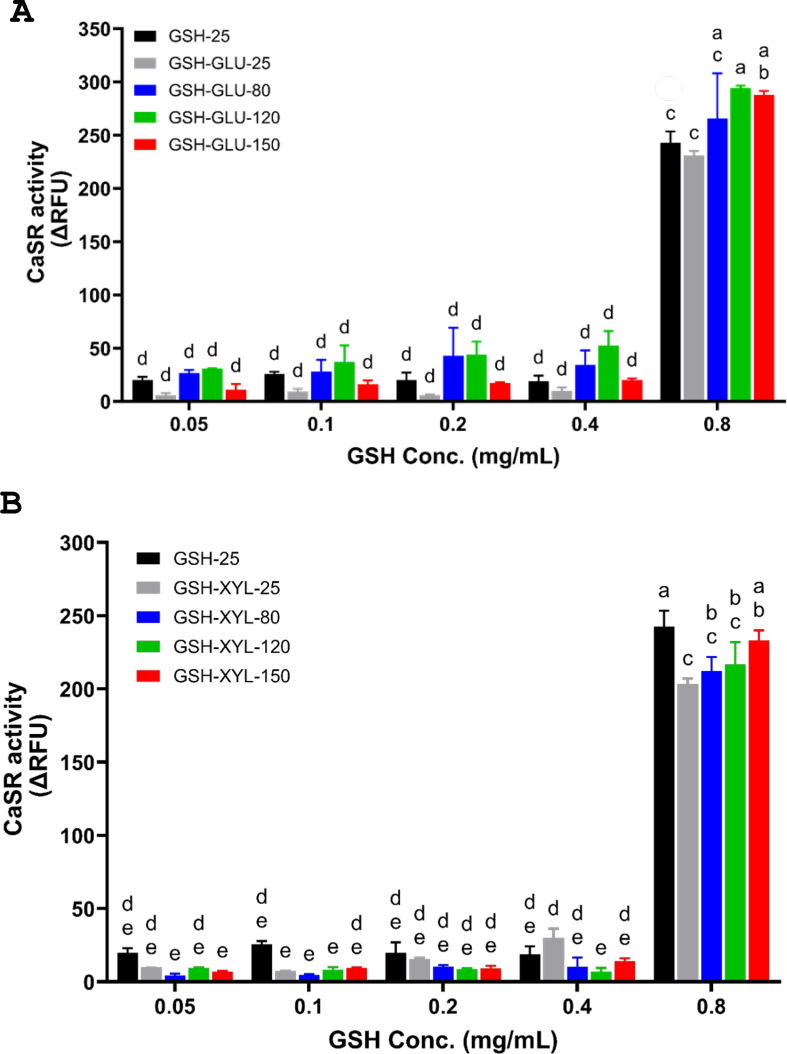
CaSR activation measured in relative fluorescence units (ΔRFU) by GSH at varying concentrations (0.05–0.5 mg/mL) when reacted with reducing sugars for 3 h at different temperatures. (A) GSH + glucose (GLU) mixtures heated at 25 °C (GSH-GLU-25), 80 °C (GSH-GLU-80), 120 °C (GSH-GLU-120), and 150 °C (GSH-GLU-150). (B) GSH + xylose (XYL) mixtures heated at 25 °C (GSH-XYL-25), 80 °C (GSH-XYL-80), 120 °C (GSH-XYL-120), and 150 °C (GSH-XYL-150). GSH alone at 25 °C (GSH-25) was included as a control in both panels. Data represent mean ± SEM from three independent experiments (n = 3). Treatments annotated with different letters are significantly different (p < 0.05). Statistical test: Two-way ANOVA with Tukey's post hoc test.

To maintain clarity and emphasize meaningful trends, only concentrations ≥0.05 mg/mL are shown in Figs. 3 and 4. Lower concentrations (0.00625–0.025 mg/mL), which yielded low ΔRFU values and no significant differences, are presented in Supplementary Fig. S2A—C.

Thermal processing of GSH was effective (*P* < 0.01) at altering the CaSR response at 0.8 mg/mL GSH at 150 °C (Fig. 3) compared to the control at 25 °C. This suggests that GSH degrades at higher temperatures potentially into small or modified peptides which may not effectively bind to and activate CaSR.

LC-MS analysis confirmed that GSH was completely degraded at 150 °C, with approximately 30 % and 5 % remaining at 80 °C and 120 °C, respectively (Suppl. Fig. S3). Previous studies by Ueda et al. (1997) identified GSH degradation into oxidized disulfide form (GSSG), pyroglutamic acid (PGA), and cyclocysteinylglycine (cycloCys-Gly) at 100 °C, regardless of pH. Higher temperatures used in this study may have further degraded PGA to other compounds. Our methods did not detect other cyclic metabolites or free amino acids that might activate CaSR. Despite GSH mostly being degraded at 150 °C, CaSR activation was observed, potentially due to cycloCys-Gly or free amino acids. This requires further investigation through metabolomics and targeted LC-MS analysis.

We also examined the Maillard reaction effect on CaSR activation when GSH was reacted with glucose (Fig. 4A) or xylose (Fig. 4B) at varying temperatures. Surprisingly, the presence of xylose at ambient 25 °C significantly decreased (*P* < 0.001) CaSR activity compared to both the GSH control and the GSH-glucose solution at the same temperature. This reduction suggests that xylose might be inhibiting the GSH response. We speculate that xylose addition to the GSH solution may interfere with allosteric binding of GSH to CaSR, reducing its activation. However, this hypothesis needs validation. To rule out direct effects of sugars alone on CaSR activation, we tested sugars at the highest concentration (100 mM) on CaSR-expressing cells and found no specific signal (Suppl. Fig. S4), aligning with literature that sugars alone do not bind and activate CaSR. This confirms that the observed effect is not due to the independent direct binding of xylose or glucose to CaSR.

When GSH (0.8 mg/mL) and glucose combined solutions were heated, the CaSR activity at 120 °C and 150 °C was significantly increased (*p* < 0.01) compared to control GSH alone and GSH-glucose solution at the ambient temperature (25 °C) (Fig. 4A) and can therefore be attributed to the presence of glucose. This effect was only evident at highest GSH concentrations of 0.8 mg/mL, suggesting that lower concentrations might not be sufficient for Maillard reaction. It has been reported that when glucose is heated to 150 °C, it degrades into organic acids such as formic, lactic, and levulinic acids. The concentration of these organic acids increases with time, with lactic acid becoming the most prominent after 5 h of heating at 150 °C (Woo, Kim, Hwang, Lee, & Jeong, 2015). When lactic acid interacts with amino acids during heating, lactoyl-peptides are formed, which have been identified as kokumi-tastants (Wu et al., 2022). It is therefore likely that lactoyl-peptide formation occurs during the thermal process and contributes to the stronger CaSR activation by MRPs.

In contrast, while xylose restored CaSR activity from 200 RFU to 235 RFU, i.e. approximately to the level of the GSH-control solution (240 RFU), it was less effective than glucose at further increasing it, with a significant effect observed only at the highest GSH concentration of 0.8 mg/mL. At lower concentrations of GSH (0.2 and 0.4 mg/mL), the reduced activity in the presence of xylose was not restored at higher temperatures (Fig. 4B).

Xylose can degrade into furfural and formic acid; however, these degradation compounds were reportedly not observed at temperatures below 150 °C and increased steadily between 150 °C and 200 °C (X. Kang et al., 2021). Xylose initially breaks down into furfural, which then further degrades into formic acids (Almeida, Bertilsson, Gorwa-Grauslund, Gorsich, & Lidén, 2009). In contrast, glucose degrades into lactic acid, which can subsequently undergo oxidation or redox reactions to form other organic acids (Shiwei & Qibao, 2018). Therefore, the degradation products of xylose, particularly lactic acid, are likely to be lower than that of glucose under these conditions, which may explain the lesser effect on CaSR activity of MRPs.

The increased browning observed at higher temperatures (120 °C and 150 °C; Fig. 2) and the generation of glutathione MRPs coincided with enhanced CaSR activation. Notably, MRPs derived from the GSH–glucose reaction was significantly more effective at activating CaSR than those from GSH–xylose (Fig. 4A, 4B). This aligns with the additional observation that Maillard reactions at 150 °C increased CaSR activity from 200 RFU (GSH–XYL–25) to 283 RFU and 235 RFU in the presence of glucose and xylose, respectively. These results suggest that the Maillard reaction not only mitigates the loss of CaSR activity due to thermal degradation of GSH but may also enhance it, particularly when glucose is involved. While GSH is already known as a kokumi-enhancing compound, our findings highlight a novel aspect: its thermal degradation products specifically MRPs can retain or regain bioactivity through interactions with reducing sugars.

Although the absolute increases in CaSR activity appear modest, they fall within a range (75–100 RFU) that, based on prior experience with various CaSR agonists, reflects meaningful receptor modulation. Such levels of activation could potentially influence kokumi-associated sensory perception in food systems, although confirmation via real food sensory evaluation is required. Importantly, while the browning intensity showed a strong correlation with total volatile peak area (*r* = 0.863), CaSR activity measured at 0.8 mg/mL did not correlate with browning intensity. Among the tested concentrations (0.05–0.8 mg/mL), only the highest dose elicited a substantial CaSR response (RFU > 200), with lower doses yielding negligible effects (<50 RFU). Although a dose-dependent increase in RFU was observed, it did not mirror browning intensity patterns.

Interestingly, xylose-treated samples exhibited greater browning and higher total volatile peak areas than glucose-treated samples, consistent with the higher reactivity of xylose. However, this did not translate into greater CaSR activation, indicating that volatile Maillard products are not the primary contributors to receptor modulation. This is further supported by the lack of correlation between browning and CaSR activity. Collectively, these results suggest that while browning and volatile generation are characteristic of the Maillard reaction, CaSR activation is more likely influenced by non-volatile Maillard products. The inclusion of volatile analysis also adds a new dimension to the study, showing that although kokumi is largely associated with non-volatile taste attributes, MRP formation can modulate aroma—particularly through sulphur-containing compounds such as DMS and DMTS. A correlation table summarizing the relationships among browning intensity, volatile peak area, and CaSR activation is provided in the Supplemental table 2.

Taken together Maillard reaction products generated from the thermal treatment of GSH and glucose at higher temperatures most likely produced compounds that act as positive allosteric modulators of the CaSR, resulting in a stronger CaSR response. Compared to GSH alone, these compounds likely demonstrate enhanced activation, indicating a higher affinity for the allosteric site. To validate these findings, molecular docking studies are necessary to explore their binding interactions with the CaSR, specifically at the binding pocket and allosteric site, and to differentiate their mechanisms from other allosteric modulators, such as gamma-glutamyl peptides, including GSH.

While GSH derived MRPs from glucose and fructose enhance beefy flavours in beef broth, xylose significantly boosts desirable beef-related sensory attributes, indicating its potent effect on flavour enhancement (Hong, Jung, Kim, Lee, & Kim, 2010; Kwon et al., 2011; Sun et al., 2019). As these studies mostly explored the contribution of aromatic compounds in modifying flavours, the contribution of non-aromatic kokumi specific taste compounds remains unclear. Our research highlights the role of non-volatile, MRPs in CaSR activation which might have potential application in producing kokumi attributes in foods as suggested elsewhere (Karangwa et al., 2016). Additionally, previous studies have identified kokumi peptides containing cysteine in MRP-xylose Maillard reaction products, which enhance desirable food attributes (Xu et al., 2018). Together, these findings underscore the promising role of MRPs in flavour enhancement through CaSR activation. However, further investigations are needed to explore the potential for GSH-glucose MRPs in generating kokumi compounds and modify flavours through their application in foods and sensory evaluation.

We note that besides allosterically binding to CaSR on the cell membrane, GSH also regulates intracellular redox status (Sies, 1999). Because GSH binds to an extracellular site on CaSR we do not expect redox interference in this assay. However, the implications of its redox function on CaSR activation require further investigation.

This study's results should be interpreted with limitations in mind. In vitro CaSR activation assays, while demonstrating activation by MRPs, do not fully capture kokumi's complex sensory attributes or the multifaceted interactions in real food systems. These assays provide insights into receptor activation but lack direct sensory validation. To confirm our hypothesis of GSH-Glucose MRPs stronger CaSR activation in food, sensory trials are essential. These will determine whether in vitro enhancements translate to enhanced kokumi attributes in real food applications, offering a clearer understanding of their culinary potential and practical relevance.

Building on the results obtained with glucose and xylose as prototype sugars, it would be valuable to explore other types of sugars, such as C6 aldoses (galactose, mannose), C6 ketoses (fructose, sorbose), and other C5 aldoses (ribose). Investigating their potential to form Maillard reaction products with glutathione, which may activate the CaSR, could reveal the formation of different reactive compounds with distinct behaviours. Investigating these interactions will enhance our understanding of how various sugars contribute to CaSR-activating MRPs.

## 4 Conclusion

This study demonstrates that CaSR activation is influenced by the thermal processing of GSH and its Maillard reaction with reducing sugars. Heating GSH alone progressively diminished CaSR activation, whereas its reaction with glucose at 150 °C restored and significantly enhanced receptor activity. Maillard products formed with xylose produced greater browning and a more diverse volatile profile; however, they exhibited lower CaSR activation compared to those derived from glucose. Formation of distinct aroma compounds, including sulphur volatiles such as DMS and DMTS, supports the potential of these reactions to modulate flavour. These findings underscore the importance of both temperature and sugar type in shaping Maillard products with sensory relevance. Given the natural occurrence of GSH and glucose in foods, this work offers practical insights for developing flavour-enhancing strategies via targeted thermal processing. However, the absence of sensory data is a limitation, and future work should include taste panel studies to confirm these effects in real food systems. Additionally, untargeted metabolomics and molecular docking studies can be used to identify novel GSH-derived Maillard compounds and their mechanisms of CaSR activation.

## CRediT authorship contribution statement

**Raise Ahmad:** Writing – review & editing, Writing – original draft, Validation, Methodology, Investigation, Formal analysis, Data curation, Conceptualization. **Santanu Deb-Choudhury:** Writing – review & editing, Resources, Project administration, Methodology, Funding acquisition, Formal analysis, Conceptualization. **Arvind Subbaraj:** Writing – review & editing, Methodology, Investigation, Formal analysis, Data curation. **Jihan Kim:** Writing – review & editing, Writing – original draft, Methodology, Investigation, Formal analysis, Data curation, Conceptualization.

## Declaration of competing interest

The authors declare that they have no known competing financial interests or personal relationships that could have appeared to influence the work reported in this paper.

## Data Availability

No data was used for the research described in the article.

## Appendix A Supplementary data

Supplementary File S1: Detailed methodologies for the analysis of volatile compounds and glutathione (GSH) peptides. Volatile compounds were extracted using solid-phase microextraction (SPME) and analysed via gas chromatography–mass spectrometry (GC–MS), with particular attention to sulphur-containing volatiles. Additional notes clarify methodological limitations and compound identification protocols. Glutathione analysis was performed using ultra-high-performance liquid chromatography coupled with quadrupole time-of-flight mass spectrometry (UHPLC-QTOF-MS), enabling identification and relative quantification of GSH and related peptides. Full instrument settings, chromatographic conditions, and data processing parameters are provided. Image 1
