# Supplementary material for: Activation of the calcium-sensing receptor by glutathione maillard products: Implications for kokumi sensation
Source: Food Chem X. 2025 Jun 3;29:102616. doi: 10.1016/j.fochx.2025.102616 (PMC12192335; doi:10.1016/j.fochx.2025.102616)
Supplement: Supplementary file 1 — Supplementary File S1: Detailed methodologies for the analysis of volatile compounds and glutathione (GSH) peptides. Volatile compounds were extracted using solid-phase microextraction (SPME) and analysed via gas chromatography–mass spectrometry (GC–MS), with particular attention to sulphur-containing volatiles. Additional notes clarify methodological limitations and compound identification protocols. Glutathione analysis was performed using ultra-high-performance liquid chromatography coupled with quadrupole time-of-flight mass spectrometry (UHPLC-QTOF-MS), enabling identification and relative quantification of GSH and related peptides. Full instrument settings, chromatographic conditions, and data processing parameters are provided. [file mmc1.docx]

**Supplementary Material**

1. **Analysis of volatile compounds by GC-MS**

Volatile compounds from the sample were extracted using solid-phase microextraction (SPME) and analysed via gas chromatography–mass spectrometry (GC–MS), following an established protocol as described by (Hutchings et al., 2025), with additional clarification below to address sulphur compound analysis and methodological precision.

For each treatment, three replicates (n = 3) were weighed (1.0 ± 0.1 g) into 20-mL clear glass screw-top headspace vials with PTFE/silicone septa (Thermo Fisher Scientific). No buffers or salts were added, and the pH no adjustment was performed.

An AOC-5000 autosampler (PAL system, CTC Analytics) was used for sample handling. Each vial was heated at 40°C for 30 min with continuous agitation at 500 rpm, followed by exposure of a 2 cm DVB/Carboxen/PDMS Stableflex SPME fiber (50/30 µm film thickness, Supelco) to the headspace for 30 min without agitation. This fiber was selected due to its broad affinity for polar and non-polar volatiles, including sulphur volatiles (Hill PG, Smith RM. Determination of sulphur compounds in beer using headspace solid-phase microextraction and gas chromatographic analysis with pulsed flame photometric detection (Hill & Smith, 2000).

Desorption of analytes was performed using a splitless injector at 240°C for 1 min, followed by a split ratio of 1:30. The fiber remained in the injector for 20 min total to ensure complete desorption.

GC–MS analysis was conducted using a Shimadzu QP-2010 Plus gas chromatography system coupled with a TQ 8040 mass spectrometer. Separation was achieved on a Shimadzu SH-624 column (60 m × 0.32 mm i.d., 1.8 µm film thickness). Helium was used as the carrier gas with a flow rate of 3.09 mL/min.

The GC oven temperature program was as follows:

- Initial temperature of 40°C (held for 2 min)
- Ramp at 5°C/min to 120°C (held for 7 min)
- Ramp at 7°C/min to 190°C (held for 9 min)
- Ramp at 10°C/min to 220°C (held for 5 min)

GC–MS interface and inlet temperatures were both maintained at 240°C, while the ion source temperature was set at 200°C. Mass spectra were acquired in Q3 scan mode across an m/z range of 35–350.

Post-run integration was automatically performed using LabSolutions software (Shimadzu), based on a slope of 750/min, 3 s peak width, and minimum peak area of 5.0E4, with T.DBL = 1000 min to account for drift.

An external standard (4-methyl-2-pentanone, 100 ppb; Merck Life Science) was injected periodically to monitor instrument performance. No internal standard was used for normalization or quantification. Consequently, reported peak intensities are presented in arbitrary units (au). While compound annotation was based on ≥90% match against the NIST14 nominal mass database (National Institute of Standards and Technology, Gaithersburg, MD, USA). Compounds with <90% similarity were excluded. No retention indices or authentic reference standards were used. Thus, all identifications should be regarded as tentative.

**Notes on Sulphur Volatiles identification**

The detection of dimethyl sulphide and dimethyl trisulfide is notable, given their role as kokumi-related flavour enhancers and Maillard reaction markers. However, sulphur volatiles are inherently challenging to detect by conventional SPME–GC–MS due to their high volatility, chemical instability, and tendency to adsorb to vial and fiber surfaces.

In this study, no specific optimizations were made for sulphur detection (e.g., no sulphur-targeted fibers, antioxidants, or low-oxygen conditions). Therefore, we acknowledge that sulphur-containing compounds may be underrepresented, and additional targeted analyses (e.g., using sulphur chemiluminescence detectors or thiol derivatization) may be needed in future work to fully capture this compound class.

Following the Reviewer’s comments, we have checked the spectral patterns of Di-methyl disulphide (DMDS) and Di-methyl trisulphide (DMTS) in the samples, and corresponding matches in the NIST database. The fragmentation spectra and sulphur isotopes confirm the identification of both DMDS and DMTS.


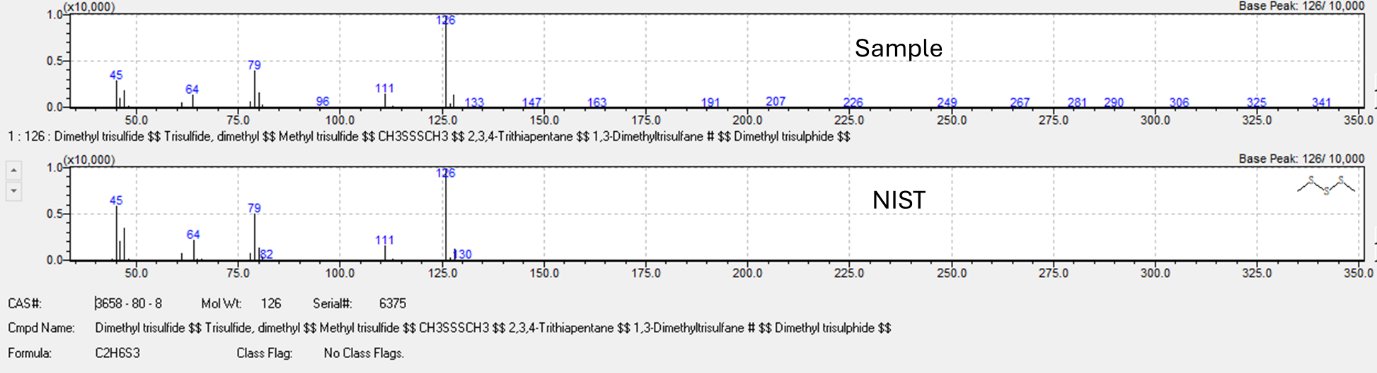


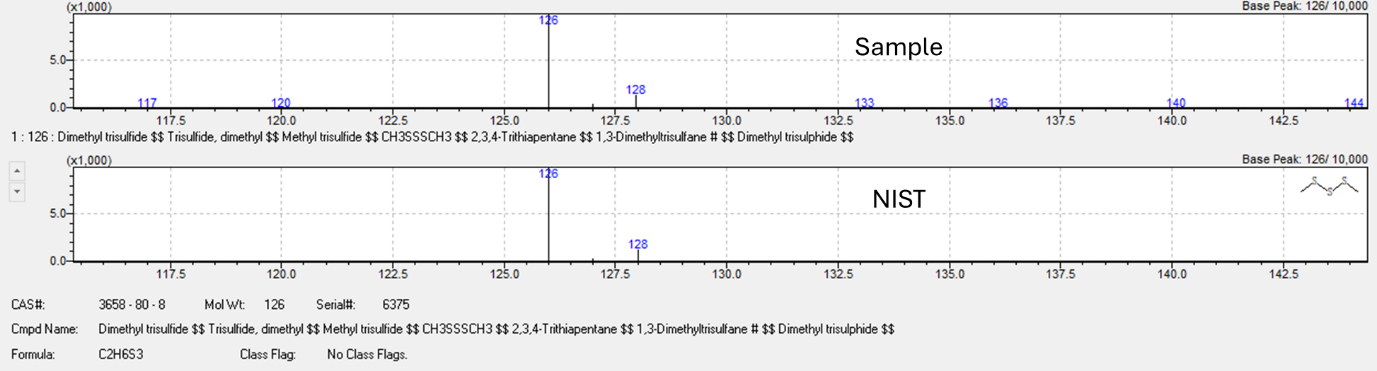


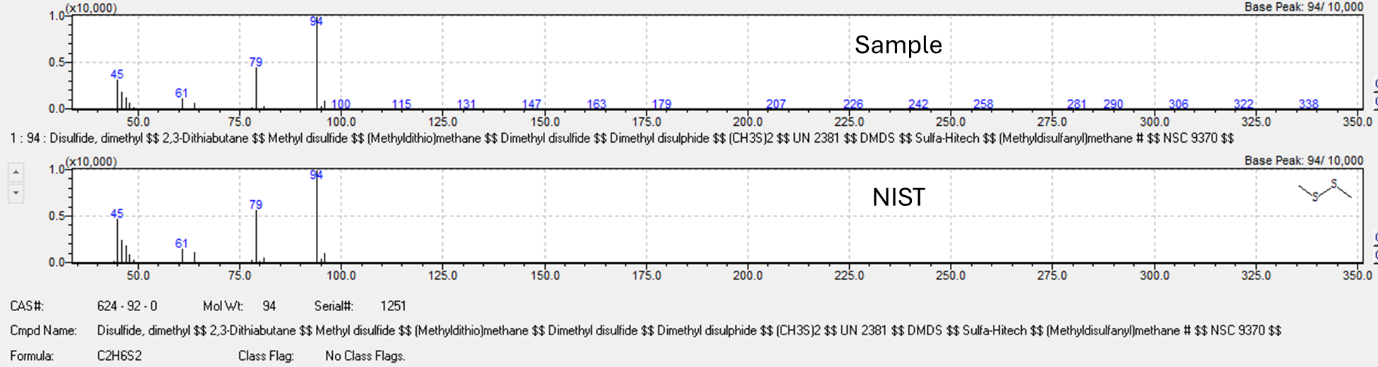


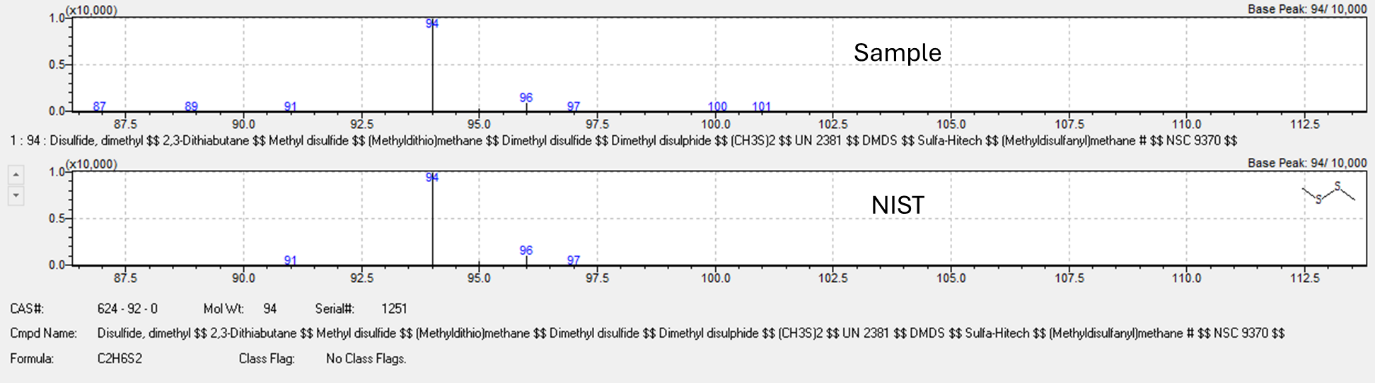


**2. Analysis of GSH by LC-MS**

Peptides in the extracts were separated by ultra-high-performance liquid chromatography (UHPLC) (Nexera X2, Shimadzu, Japan) coupled to a LCMS-9030 quadrupole time-of-flight (Q-TOF) mass spectrometer equipped with an [electrospray ionization](https://www.sciencedirect.com/topics/food-science/electrospray-ionization) source (Shimadzu, Japan). Sample (10 µL) was injected into a normal phase Ascentis® Express HILIC UHPLC column (2.1 × 100 mm, 2 µm particle size; Sigma, USA) and eluted at 30 °C over a 20-minute gradient with a flow rate of 400 µL/minute. The mobile phase solvent A was 10 mM ammonium formate in water and solvent B was acetonitrile with 0.1% formic acid. The solvent gradient program started at 97% solvent B from 0 to 0.5 mins, decreased to 70% within 11.5 mins and further to 10% from 11.5 to 13.5 mins, held at 10% for 1.5 mins, increased to 97% B within 1 min and held at that concentration until the end of the elution run.

An authentic glutathione (GSH) standard was run under identical conditions (Sigma-Aldrich Chemicals Co., MO USA) to determine experimental fragmentation patterns, and corresponding matches in a public domain mass spectral database (METLIN). *m/z* 128.0350, representative of the deprotonated glutathione, was designated as the diagnostic fragment of the [glutamic acid](https://www.sciencedirect.com/topics/food-science/glutamic-acid) residue in all dipeptides. Glutathione degradation was monitored using full-scan (m/z 55–1100) and multiple reaction monitoring (MRM) modes in negative ionization mode. The spray voltage was set to −3.0 kV, with a collision energy of 20 ± 10 V and a loop time of 1.0 s across 25 events. Ion source parameters were optimized as follows: nebulizing gas flow at 3.0 L/min, heating gas flow at 10.0 L/min, interface temperature at 300 °C, drying gas flow at 10.0 L/min, desolvation line temperature at 250 °C, and heat block temperature at 400 °C. Samples were analysed under similar conditions.

Peak detection and area integration were performed using LabSolutions Insight software (version 3.50SP2; Shimadzu, Japan). Relative quantification was based on comparisons of peak areas between sample groups for each analyte, as determined from extracted ion chromatograms.

**References**

Hutchings, S. C., Deb‐Choudhury, S., Subbaraj, A. K., Guerrero, L., Torrico, D. D., Ham, E. E., & Realini, C. E. (2025). Characterizing the odor of New Zealand native plants using sensory analysis and gas chromatography–mass spectrometry. *Journal of Food Science*, *90*(2), e70050.

Hill, P. G., & Smith, R. M. (2000). Determination of sulphur compounds in beer using headspace solid-phase microextraction and gas chromatographic analysis with pulsed flame photometric detection. *Journal of chromatography. A*, *872*(1-2), 203–213. <https://doi.org/10.1016/s0021-9673(99)01307-2>
